# Supplementary material for: Creation of a long-acting nanoformulated dolutegravir
Source: Nat Commun. 2018 Feb 6;9:443. doi: 10.1038/s41467-018-02885-x (PMC5799307; doi:10.1038/s41467-018-02885-x)
Supplement: Supplementary file 1 — Supplementary Information [file 41467_2018_2885_MOESM1_ESM.pdf]

Creation of a Long-Acting Nanoformulated Dolutegravir

Sillman *et al.*

Supplementary Figure 1

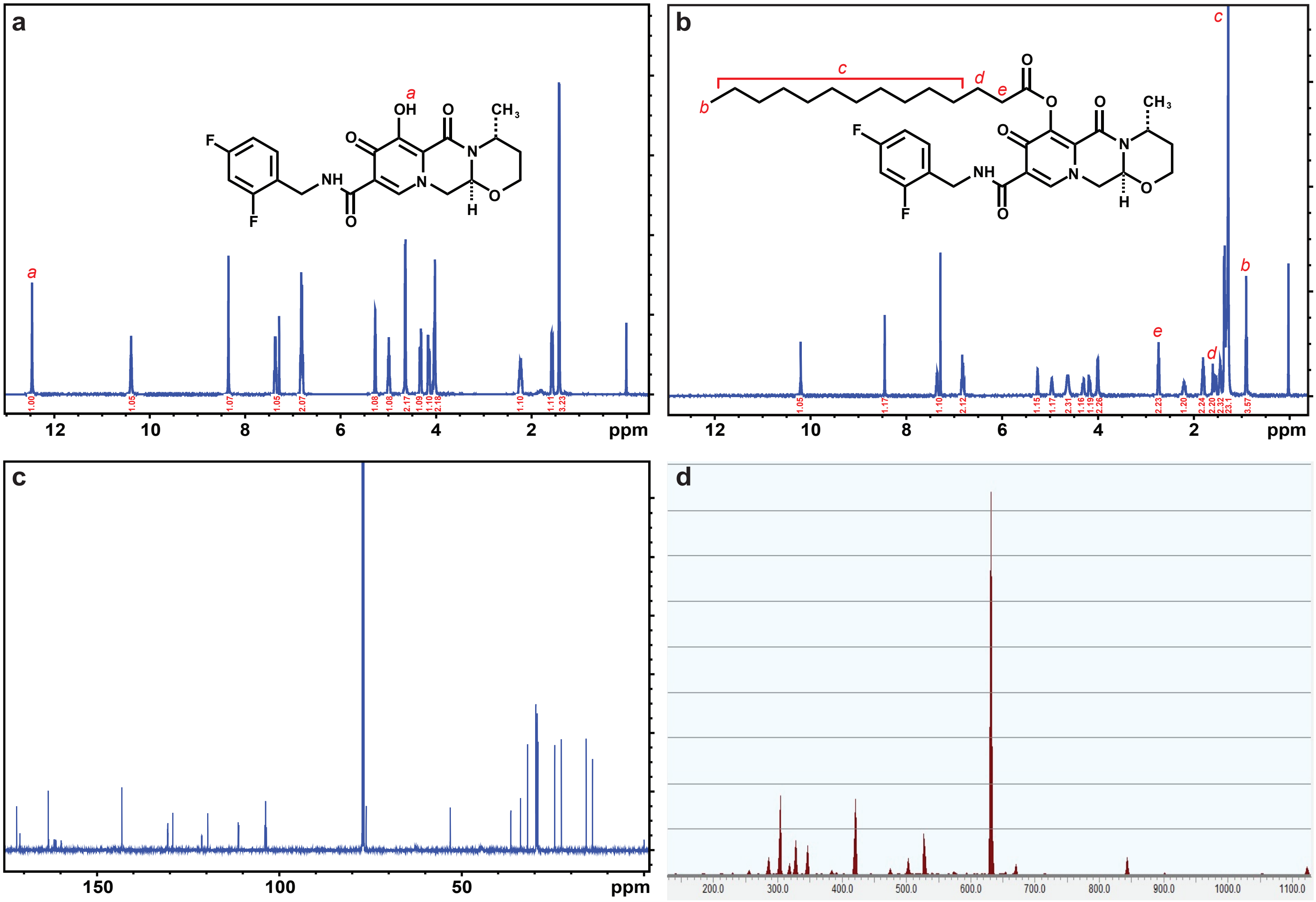

Supplementary Figure 1. Chemical characterization of MDTG. Proton nuclear magnetic resonance (^{1}H NMR) spectral analysis of DTG (**a**) shows the loss of the peak corresponding to the 7-hydroxyl proton of DTG (*a*), while the spectrum of MDTG (**b**) illustrates several unique peaks (*b*-*e*) that correspond to the protons present on the covalently linked myristic acid moiety. (**c**) Carbon nuclear magnetic resonance (^{13}C NMR) spectral analysis of MDTG shows all 34 carbon atoms present on the modified drug. (**d**) Positive electrospray ionization mass spectroscopy (ESI-MS) analysis shows the exact mass of MDTG to be 629.33 (100%).

Supplementary Figure 2

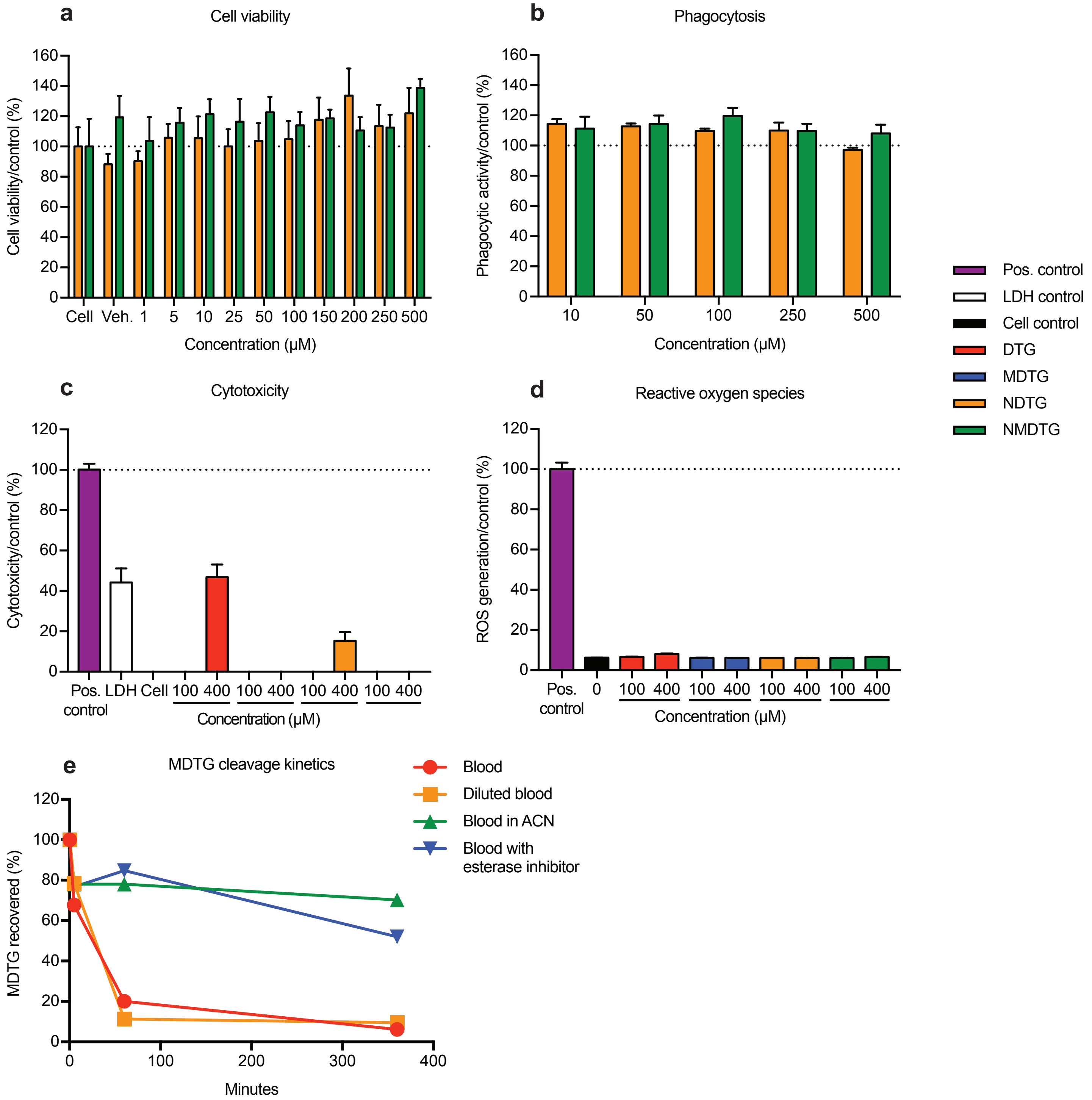

Supplementary Figure 2. MDTG cleavage kinetics and MDM function after NDTG and NMDTG treatments. (**a**) Cell viability was assessed in MDMs by MTT assay 6 hours after NDTG or NMDTG treatment over a range of concentrations (1 - 500 μM) for 8 hours. Results were normalized to untreated control cells. (**b**) Phagocytic activity was assessed in MDMs by Vybrant™ phagocytosis assay after NDTG or NMDTG treatment over a range of concentrations (10 - 500 μM) for 8 hours. Results were normalized to untreated control cells. (**c**) Cytotoxicity was assessed in MDMs by LDH assay 24 hours after 100 and 400 μM NDTG or NMDTG treatment for 8 hours. Results were normalized to cell lysate control. (**d**) Reactive oxygen species generation was assessed in MDMs by DCFDA assay after 100 and 400 μM NDTG or NMDTG treatment for 2 hours. Results were normalized to DCFDA-treated positive control cells. (**e**) Cleavage of MDTG was assessed *ex vivo* in whole mouse blood. Known concentrations of MDTG were spiked into blood, blood diluted 10X in PBS, blood that was immediately added to acetonitrile (ACN), or blood spiked with an esterase inhibitor cocktail. At various time points samples were collected and analyzed for MDTG levels by UPLC-MS/MS. All results are shown as the mean ± SEM of at least 3 biological replicates.

Supplementary Figure 3

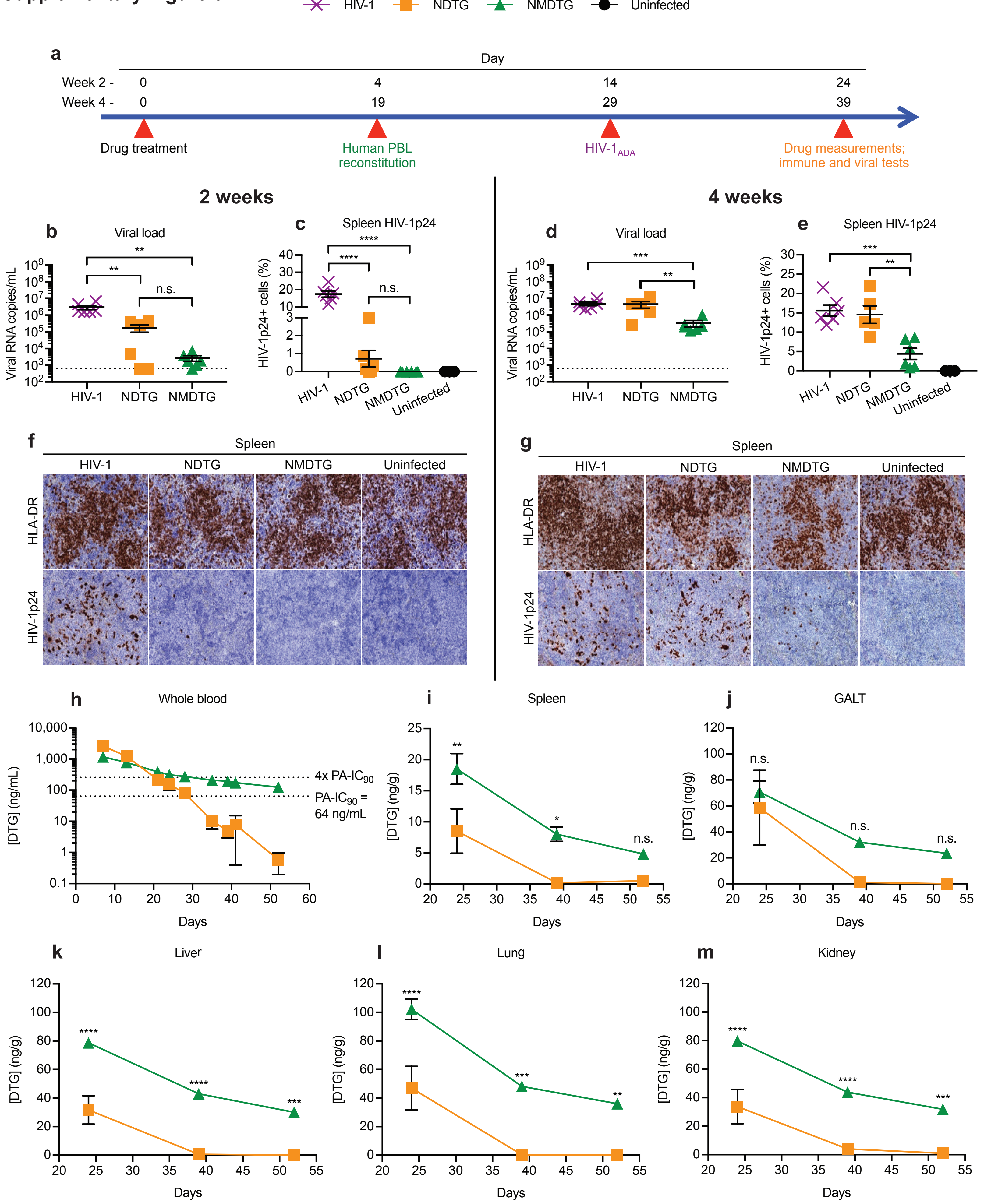

Supplementary Figure 3. Viral restriction in PBL reconstituted mice. NSG mice were treated according to the scheme illustrated in (**a**). Uninfected mice without treatment served as negative controls. HIV-1-infected mice without treatment served as positive controls. Plasma viral load was measured at terminal euthanasia after HIV-1 challenge (**b**) two-weeks and (**d**) four-weeks post drug treatment. ** $P < 0.01$, *** $P < 0.001$. Spleen sections were stained for HIV-1p24 and HLA-DP/DQ/DR, and HIV-1p24+ and HLA-DP/DQ/DR+ cells were quantified. Results are represented as percent HIV-1p24/HLA-DP/DQ/DR+ cells after HIV-1 challenge (**c**) two-weeks and (**e**) four-weeks post drug treatment. ** $P < 0.001$, *** $P < 0.001$, **** $P < 0.0001$. Representative HLA-DP/DQ/DR (top) and HIV-1p24 (bottom) staining (brown) of spleen sections quantified after HIV-1 challenge (**f**) two-weeks and (**g**) four-weeks post drug treatment. (**h**) Blood DTG concentrations were analyzed by UPLC-MS/MS. Dotted lines indicate the PA-IC_{90} (64 ng/mL) and four-times the PA-IC_{90} (256 ng/mL). (**i-m**) DTG concentrations were analyzed by UPLC-MS/MS in spleen (**i**), GALT (**j**), liver (**k**), lung (**l**), and kidney (**m**). Drug levels were tested at days 24, 39, and 52. * $P = 0.0280$, ** $P < 0.\ 01$, *** $P < 0.\ 001$, **** $P < 0.0001$. Results are shown as the mean ± SEM of 5-6 biological replicates (Uninfected group – n = 3). Findings obtained from **b-e** were analyzed by one-way ANOVA with Bonferroni's multiple comparison tests. Results from **i-m** were analyzed by two-tailed ANOVA with Bonferroni's multiple comparison tests.

Supplementary Figure 4

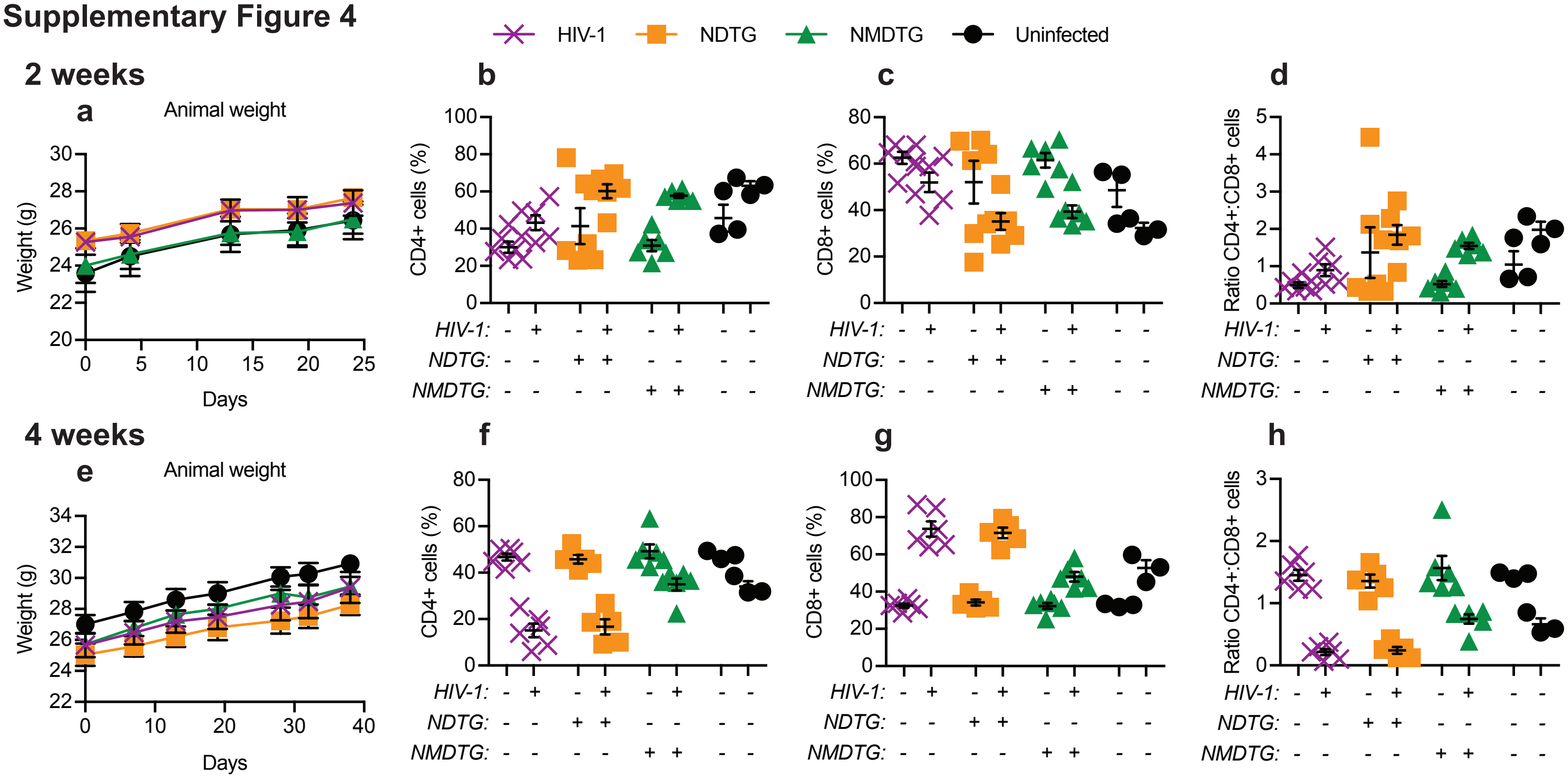

Supplementary Figure 4. Animal weights and flow cytometry tests of PBL reconstituted mice. (**a,e**) Animal weights were monitored throughout the length of the study to assess animal health. Cell phenotyping analysis of human CD4+ (**b,f**) and CD8+ (**c,g**) T cell populations determined from CD3+ lymphocytes in the total CD45+ cell population by flow cytometry. (**d,h**) CD4:CD8+ cell ratios were calculated. All groups were analyzed before infection and at the time of terminal euthanasia. Results are shown for the two-week (**a-d**) and four-week (**e-h**) viral challenges. Uninfected mice without treatment served as negative controls. HIV-1-infected mice without treatment served as positive controls. Results are shown as the mean ± SEM of 5-6 biological replicates (Uninfected group – n = 3).

Supplementary Table 1 – Pharmacokinetic parameters

PK Parameters	NDTG		NMDTG	
	Average	SEM	Average	SEM
**λ_Z (1/hr)**	0.01153	0.00088	0.00211	0.00006
**$t_{1/2}$ (hr)**	61.9	4.7	330.4	8.9
**AUC_{last} (hr*ng/mL)**	894,733.4	33,830.6	934,923.7	39,285.3
**$AUC_{0-\infty}$ (hr*ng/mL)**	895,196.6	33,747.3	977,470.7	41,805.5
**AUC % Extrapolation**	0.05	0.04	4.34	0.23
**V_β/F (L/kg)**	4.55	0.44	22.10	0.94
**CL/F (L/hr/kg)**	0.051	0.002	0.046	0.002
**$MRT_{0-\infty}$**	104.2	3.2	348.8	7.4

Dose = 45 mg/kg DTG-eq. Non-compartmental analysis N = 6 Whole blood NDTG = D1-28, NMDTG = D1-56

λ_Z – terminal elimination rate constant

AUC_{last} – AUC 0 hr to last time point

$AUC_{0-\infty}$ – AUC 0 hr to infinity

V_β/F – apparent volume of distribution

CL/F – apparent total clearance

MRT – mean residence time
